# Supplementary material for: Acceptance and compliance with micronutrient powder (MNP) among children aged 6–23 months in northern Nigeria
Source: PLOS Glob Public Health. 2022 Oct 17;2(10):e0000961. doi: 10.1371/journal.pgph.0000961 (PMC10022258; doi:10.1371/journal.pgph.0000961)
Supplement: S1 Dataset — (ZIP) [file pgph.0000961.s008.zip › S8_Dataset/Spot Checks.zip!/Spot Checks/11072015_A_GU_ID_2_SC a.pdf]

SPOT CHECK RECORDING FORM

State: Adamawa **LGA: ____Song____ Community: _Gombawa_**

Name of caregiver (female): Suraiya Ibrahim

Name or caregiver (male): ………………………

Sex of index child: _Male___ Age of index child (in months): _ 24 months__________

Spot Check Information

1. **Date of MNP distribution to household** (circle option below based on LGA name above)**:**

- **Kebbi:** BirninKebbi&Jega LGA: **June 16**
- **Kebbi:** Danko-wasagu: **June 17**
- **Adamawa:**Song, Ganye: **June 18**
- **Adamawa:**Guyuk **June 19**

2. **Date of Spot Check Observation: ____11/7/2015_____**

3. **Number of sachets remaining**(Ask the caregiver to show you and count the specific number of sachets remaining)**:**

36

4. **Number of sachets that should remain using one MNP sachet per day** (Analyst only)**:**

39

5. **Number of sachets remaining / number of sachets should be remaining**(Analyst only)**:**

_____36____ / ___39______ = __92.3__ %

Probes

Question 1. You have **XX** MNP sachets remaining. Explain how easy or difficult it was to use the MNP product on a daily basis for your child 6 – 23 months? (Circle one answer)

**Very Difficult**	**Difficult**	**Easy**	**Very Easy**

Question 2. Describe any specific challenges that you faced that made the MNP difficult to use on a daily basis?

No challenges.

Question 3. Describe specific aspects of the MNP product that you or your child enjoyed that made it easier to use on a daily basis?
MNP make my child to be eaten food and has increase his weight. He doesn't fall sick and the MNP is protecting him from falling sick.

Question 4. What questions do you have about the MNP based on your experience using it?

- When the MNP is mix with pap and the child did not take it and the mix MNP and pap change colour and on the pamphlet the instructions was that it should not be given to the child after 30 minutes. The elder sister took the mix pap, I hope it will not cause problem to her.

Question 5. What specific recommendations do you have that we can do as a program to make the MNP easier to use on a daily basis?

The MNP is very good to my child because some of my children before they start growing teeth they usually purge but surprisingly with the use of MNP no purging again because of teething.
